# Nanostructured Lipid Carriers (NLCs) for Oral Peptide Drug Delivery: About the Impact of Surface Decoration

**DOI:** 10.3390/pharmaceutics13081312

**Published:** 2021-08-22

**Authors:** Iram Shahzadi, Andrea Fürst, Patrick Knoll, Andreas Bernkop-Schnürch

**Affiliations:** Center for Chemistry and Biomedicine, Department of Pharmaceutical Technology, Institute of Pharmacy, University of Innsbruck, Innrain 80-82, A-6020 Innsbruck, Austria; iramuvas@gmail.com (I.S.); Andrea.Fuerst@student.uibk.ac.at (A.F.); Patrick.Knoll@uibk.ac.at (P.K.)

**Keywords:** insulin, nanostructured lipid carriers, pepsin, pancreatin, proteolysis

## Abstract

This study was aimed to evaluate the impact of surfactants used for nanostructured lipid carriers (NLCs) to provide enzymatic protection for incorporated peptides. Insulin as a model peptide was ion paired with sodium dodecyl sulfate to improve its lipophilicity. Three NLC formulations containing polyethylene glycol ester (PEG-ester), polyethylene glycol ether (PEG-ether), and polyglycerol ester (PG-ester) surfactants were prepared by solvent diffusion method. NLCs were characterized regarding particle size, polydispersity index, and zeta potential. Biocompatibility of NLCs was assessed on Caco-2 cells via resazurin assay. In vitro lipolysis study was performed using a standard lipid digestion method. Proteolytic studies were performed in simulated gastric fluid containing pepsin and simulated intestinal fluid containing pancreatin. Lipophilicity of insulin in terms of log P_octanol/water_ was improved from −1.8 to 2.1. NLCs were in the size range of 64–217 nm with a polydispersity index of 0.2–0.5 and exhibited a negative surface charge. PG-ester NLCs were non-cytotoxic up to a concentration of 0.5%, PEG-ester NLCs up to a concentration of 0.25% and PEG-ether NLC up to a concentration of 0.125% (*w*/*v*). The lipolysis study showed the release of >90%, 70%, and 10% of free fatty acids from PEG-ester, PG-ester, and PEG-ether NLCs, respectively. Proteolysis results revealed the highest protective effect of PEG-ether NLCs followed by PG-ester and PEG-ester NLCs for incorporated insulin complex. Findings suggest that NLCs bearing substructures less susceptible to degrading enzymes on their surface can provide higher protection for incorporated peptides toward gastrointestinal proteases.

## 1. Introduction

Advances in medicinal chemistry and biotechnology have resulted in the emergence of numerous therapeutic peptides representing one of the fastest-growing class of new active pharmaceutical ingredients. Most of these drugs, however, have to be administered via the parenteral route, and especially in case of certain chronic conditions such as diabetes or cancer, therapeutic peptides exhibiting a short biological half-life have to be administered frequently, causing a substantial drop in compliance. The development of advanced drug delivery systems enabling administration via alternative routes and, in particular, the oral route is therefore highly on demand [1]. Among such advanced drug delivery systems, lipid-based nanocarriers have shown considerable potential as they help to overcome the two most important barriers for oral peptide delivery: (i) the enzymatic barrier and (ii) the absorption barrier of the gastrointestinal tract (GIT) [2]. Furthermore, lipid-based nanocarriers can be designed by using biocompatible and biodegradable lipid excipients and consequently do not exert toxic effects [3]. Since the first oral lipid-based nanocarrier formulation for the highly lipophilic peptide cyclosporine entered the global market in the 1980s, numerous types of lipid-based nanocarriers such as nanoemulsions [4], liposomes [5], solid lipid nanoparticles (SLNs) [6], and nanostructured lipid carriers (NLCs) [7] have been employed for oral peptide drug delivery. As it turned out difficult to control peptide drug release out of liquid lipid-based nanocarriers [8], SLNs and NLCs moved into the limelight of research. Since solid lipids are used instead of oils and diffusion of peptide drugs in a solid lipid is considerably lower compared to an oily phase, their release can be controlled to a much higher extent [9]. NLCs were developed to overcome the problem of drug expulsion from SLNs upon storage due to crystallization and phase transition of solid lipids [10]. Few research studies provide even evidence for the successful incorporation of peptide drugs in NLCs [7,11]. Glargine insulin, for instance, was successfully loaded in NLCs after ion pairing with sodium dodecyl sulfate (SDS). Due to the encapsulation in the lipophilic phase, a significant decrease in blood glucose levels in diabetic rats was shown after oral administration of NLCs [11]. The majority of lipid excipients used for the design of NLCs, however, is susceptible to degradation by gastrointestinal lipases. Chain length and number of cleavable linkages in lipids as well as the type of surfactant in NLCs may have an impact on their lipolysis and the subsequent degradation of incorporated peptides by proteases [12].

In the case of lipid-based nanocarriers, surfactants remain on the lipid/water interface and are therefore easily accessible for degrading enzymes. The type and quantity of cleavable substructures on the surface of lipid-based nanocarriers have, therefore, likely a great impact on their stability toward enzymatic degradation. So far, however, no evidence for this theory has been provided at all. So the study was aimed to evaluate the impact of NLC surface decorations using various surfactants on their protective effect toward incorporated peptides. Insulin (INS) was used as a model peptide and was loaded in NLCs after lipidization with sodium dodecyl sulfate (SDS). NLC formulations underwent basic pharmaceutical characterization and were then evaluated in vitro for toxicity and their potential to provide protection against gastrointestinal enzymes.

## 2. Materials and Methods

### 2.1. Materials

Human recombinant insulin, sodium dodecyl sulfate, stearic acid, pepsin from porcine gastric mucosa (≥250 units/mg solid), pancreatin from porcine pancreas (8 × USP) and resazurin were purchased from Sigma-Aldrich Chemie GmbH, Vienna, Austria. Peceol (glycerol mono-oleate type 40) and Kolliphor RH 40 were a gift from Gattefossé (Lyon, France). Brij L23, Tegosoft PC41, and Tegosolve 90MB were obtained from Evonik Nutrition & Care GmbH, Essen, Germany. Oleic acid was purchased from Gatt-Koller, Absam, Austria. All other reagents, chemicals, and solvents used were of analytical grade and obtained from commercial sources.

### 2.2. HPLC Method

Insulin was quantified via an already established HPLC method as described by Nazir et al. [13]. Nucleosil C18 100-5 column (Macherey-Nagel GmbH, Düren, Germany) (5 μm, 4.6 × 250 mm) was used as stationary phase. As mobile phase 0.1% (*v*/*v*) trifluoroacetic acid (Sigma-Aldrich Chemie GmbH, Vienna, Austria) (TFA) was employed as eluent A and acetonitrile (VWR International, Fontenay-sous-Bois, France) as eluent B at a flow rate of 1 mL/min and a temperature of 40 °C. Gradient elution method was used as follows: 0–12 min: 70–45% A and 30–55% B, 12–14 min: 45–70% A, and 55–30% B. Sample injection volume was 20 µL, and analysis was performed at 214 nm.

### 2.3. Formation of Hydrophobic Ion Pairs of Insulin

Hydrophobic ion pairs of insulin with sodium dodecyl sulfate (SDS) as counter ion were prepared using an already established method [14]. Positive charges on insulin were induced by dissolving the peptide in 0.01 M HCl (Sigma-Aldrich Chemie GmbH, Vienna, Austria) at the concentration of 1 mM. SDS was dissolved in demineralized water at increasing concentrations (1–10 mM). For the formation of insulin ion-pair complexes, SDS solution was added dropwise to insulin solution (1/1, *v*/*v*) while continuously stirring at 300 rpm at 25 °C. Ion-pair complexes formed immediately upon the addition of SDS solution resulting in white precipitates. Mixtures were further stirred under the same conditions for 4 h. Finally, INS-SDS complexes were isolated by centrifugation for 10 min at 13,400 rpm and washed three times with demineralized water. In the following, they were freeze-dried and stored at −20 °C until further use.

For the determination of precipitation efficiency at each molar ratio, supernatants were analyzed for remaining insulin using the HPLC method as described above. Precipitation efficiency (PE) was calculated using the following equation:

(1) $$\mathrm{PE}\;\left(\%\right)=100-\left(\frac{\mathrm{INS}\;\mathrm{concentration}\;\mathrm{after}\;\mathrm{HIP}}{\mathrm{INS}\;\mathrm{concentration}\;\mathrm{before}\;\mathrm{HIP}}\times 100\right)$$

Results of percentage precipitation efficiency were plotted against SDS: insulin molar ratios.

### 2.4. Determination of Lipophilicity

Log P_octanol/water_ of INS and INS-SDS complexes was determined using a previously described method with minor modification [13]. Briefly, 1 mg of each product was added to 1 mL of octanol/water (1:1) (Sigma-Aldrich Chemie GmbH, Vienna, Austria). The mixtures were incubated at 37 °C while stirring at 500 rpm for 12 h. Thereafter, samples were centrifuged at 13,400 rpm for 10 min. Aliquots of 100 μL were withdrawn from each phase (octanol and water) and analyzed via HPLC after dilution with 300 μL of methanol (Sigma-Aldrich Chemie GmbH, Vienna, Austria) containing 0.1% (*v*/*v*) TFA. Log P was calculated according to the following equation:

$$\mathrm{Log}\;P=\mathrm{Log}\left(\frac{\mathrm{Concentration}\;\mathrm{of}\;\mathrm{INS}\;\mathrm{in}\;\mathrm{octanol}\;\mathrm{phase}}{\mathrm{Concentration}\;\mathrm{of}\;\mathrm{INS}\;\mathrm{in}\;\mathrm{water}\;\mathrm{phase}}\right)$$

### 2.5. Preparation of NLC Formulations

Three NLC formulations with different surfactants were prepared, first formulation with PEG-ester surfactant, second with PEG-ether and third with poly glycerol ester surfactants. The lipid and surfactant components of each NLC formulation are listed in Table 1.

NLCs with or without INS-SDS complex were prepared using a method having been reported previously [15]. Briefly, 40 mg of solid lipid (Sigma-Aldrich Chemie GmbH, Vienna, Austria) and 16 mg of liquid lipid with or without INS-SDS complex (10 mg) were dissolved in 500 µL of ethanol and acetone (Donauchem GmbH, Vienna, Austria) (1:1) mixture at 50 °C and 400 rpm in a thermomixer. The resulting solution was dispersed in 5 mL of aqueous surfactant solution (1.0% *w*/*v*) at 50 °C under continuous stirring. After 5 min, the dispersion was quickly cooled in an ice bath and kept on stirring until room temperature was reached. Formed NLCs were separated by centrifugation at 13,400 rpm for 20 min, re-dispersed in aqueous surfactant solution (1.0% *w*/*v*) solution, freeze-dried, and stored at 4 °C until further use. Supernatants were analyzed by HPLC to determine entrapment efficiency (EE) using the following equation:

$$\mathrm{EE}\;\left(\%\right)=\frac{\mathrm{Remaining}\;\mathrm{INS}}{\mathrm{Total}\;\mathrm{intial}\;\mathrm{INS}}\times 100$$

### 2.6. Basic Characterization of NLCs

The average NLCs particle size, PDI, and zeta potential were analyzed by photon correlation spectroscopy (PCS) technique using Zetasizer Nano-ZSP (Malvern Instruments, Worcestershire, UK) at 37 °C using 1:100 dilution of each NLC in purified water [13]. All measurements were performed in triplicate and reported in the form of means ± standard deviation (SD).

### 2.7. Cytotoxicity Study

Blank and INS-SDS loaded NLCs were investigated regarding their cytotoxic potential on the Caco-2 cell line via resazurin assay [16]. For this purpose, Caco-2 cells were transferred to 24-well plates at a seeding density of 2.5 × 10^4^ cells per 0.5 mL of red MEM (minimum essential medium) in each well. Red MEM used as cell growth medium contained antibiotics (100 units penicillin, 0.1 mg streptomycin/L) (PAN-Biotech GmbH, Aidenbach, Germany) and 10% *v*/*v* fetal bovine serum (Catus Biotech GmbH, Tutzing, Germany). Cells were cultured for two weeks at 37 °C under 95% relative humidity and 5% CO_2_. During the cell culture period, growth medium was replaced on alternate days. For the experiment, test samples of INS-SDS were prepared by suspending them in the concentration range of 0.0625 to 1% (*w*/*v*) in sterile HBS (Thermo Fisher GmbH, Kandel, Germany) (HEPES buffered saline) pH 7.4. HBS only was used as a positive control, and Triton X-100 (Sigma-Aldrich Chemie GmbH, Vienna, Austria) (0.2%, *v*/*v*) in HBS was used as a negative control.

For the experiment, growth medium from all wells of the cell culture plate was removed, and cells were washed three times with sterile 0.01 M phosphate-buffered saline (Biochrom GmbH, Berlin, Germany) (PBS) pH 7.4 preheated at 37 °C. To individual wells of cell culture plate, 0.5 mL of each test sample or control were added and incubated under conditions as described above for 4 h. Thereafter, all test samples and controls were removed from wells and washed using preheated PBS pH 7.4 at 37 °C (3 × 0.5 mL). Thereafter, 2.2 mM resazurin (0.5 mL) was added to each well, and cells were again incubated under the same conditions for 3 h. The fluorescence of the supernatant from each well was measured at the excitation wavelength of 540 nm and an emission wavelength of 590 nm. Cell viability was calculated by putting the fluorescence values (Flu) of test samples, negative and positive controls, in the following equation:

$$\mathrm{Cell}\;\mathrm{viability}\;\left(\%\right)=\left(\frac{\mathrm{Flu}\;\left(\mathrm{Test}\right)-\mathrm{Flu}\;\left(\mathrm{Negative}\right)}{\mathrm{Flu}\;\left(\mathrm{Positive}\right)-\mathrm{Flu}\;(\mathrm{Negative})}\right)\times 100$$

### 2.8. In Vitro Lipolysis Study

#### 2.8.1. Preparation of Digestion Medium

The lipid digestion medium was prepared as described previously [17]. The fasted state phospholipid/bile salt mixed micelles were prepared in the following sequence: egg lecithin (1.25 mM) was dissolved in chloroform and followed by evaporation under vacuum to form a thin film of lecithin on the walls of a round-bottom flask. Sodium taurodeoxycholate, (5 mM), NaCl (150 mM) and CaCl_2_ 2H_2_O (5 mM) in digestion buffer (50 mM trizma maleate pH 7.5) were added (Sigma-Aldrich Chemie GmbH, Vienna, Austria). The mixture was stirred at room temperature using a magnetic stirrer for 12 h to obtain a transparent (light yellow) micellar solution. Pancreatin extracts were freshly prepared by stirring 1 g of porcine pancreatin powder in 5 mL of digestion buffer for 15 min, followed by centrifugation at 11,000 rpm for 15 min. The supernatant was collected and stored on ice until use.

#### 2.8.2. Digestion Experimental Procedure

The progress of lipid digestion was monitored for 60 min by using a pH-stat titration method according to the lipolysis protocol as described by Tan et al. [17]. Test samples were prepared by dispersing 100 mg of each NLC formulation in buffered micellar solution (9 mL) via stirring at 37 °C for a few min. The pH of test dispersion was adjusted to 7.5 using either NaOH or HCl (0.5 M). In order to start lipolysis, pancreatin extract (1 mL) was added. The drop in pH due to the release of free fatty acids (FFA) mediated by lipid digestion was monitored. The decrease in pH during the experiment was readjusted to 7.5 with 0.5 M NaOH. The total consumed volume of NaOH during the experiment was used to calculate FFA release based on the stoichiometric reaction ratio of 1:1. Control experiment without NLCs in micellar solution was conducted in order to take the drop in pH due to components other than those of NLCs into account. The volume of NaOH used in the control experiment was subtracted from the NaOH volume consumed during each NLC lipolysis experiment.

### 2.9. Degradation Studies

For degradation studies with pepsin, simulated gastric fluid (SGF) was prepared according to a method described by Wang et al. with minor modifications [18]. Sodium chloride solution (100 mL) was prepared in a concentration of 0.02% (*w*/*v*), and pH was adjusted to 1.2 with HCl (1 M). Thereafter, pepsin (0.32 g) was added, and the mixture was shaken gently until completely dissolved. Degradation studies were performed by adding 100 µL of each enzyme solution to 100 µL of each NLC dispersion in reaction buffer and incubated in a thermomixer at 37 °C. As a control dispersion of INS-SDS complex in reaction buffer was used. The concentration of INS-SDS in each NLC dispersion and control was 1 mg·mL^−1^. The enzymatic reaction was stopped at predetermined time points by the addition of 100 µL of 0.002 M NaOH to the reaction mixture. The samples were centrifuged at 13,400 rpm for 5 min. Both precipitates and supernatants were analyzed to determine insulin at various time points by HPLC. For analysis of precipitates, they were washed with demineralized water (100 µL × 3), dissolved in 200 µL of ethanol, and analyzed in the same way as supernatants after the addition of 100 µL of 0.002 M NaOH. Results were expressed as a percentage of remaining insulin (supernatant + sediment) at each time point.

For degradation studies with pancreatin, simulated intestinal fluid (SIF) was prepared according to a method described by Wang et al. with minor modifications [18]. Monobasic potassium phosphate (0.68 g) was dissolved in 25 mL of demineralized water, and NaOH was added to adjust pH to 6.8. To this, 0.4 g of pancreatin was added, shaken gently until dissolved, and the volume was adjusted to 100 mL with demineralized water. Pancreatin was added after adjusting the pH of the solution to 6.8 to avoid precipitation of the enzyme. Test samples of NLCs were prepared by dispersing them in reaction buffer. The concentration of INS-SDS in each sample was 1 mg·mL^−1^. Control was prepared by dispersing an equivalent amount of INS-SDS complex in reaction buffer as in test samples. The degradation studies were performed by the addition of 100 µL of each enzyme solution to 100 µL of each NLC dispersion and control. Mixtures were incubated at 37 °C in a thermomixer. The enzymatic reaction was stopped at predetermined time points by the addition of 100 µL of 0.01 M HCl. The samples were centrifuged at 13,400 rpm for 5 min. Both precipitates and supernatants were analyzed to determine insulin at various time points by HPLC. For analysis of precipitates, they were washed with demineralized water (100 µL × 3), dissolved in 200 µL of ethanol, and analyzed in the same way as supernatants after the addition of 100 µL of 0.01 M HCl. Results were expressed as a percentage of remaining insulin at each time point.

### 2.10. Data Analysis

Data are shown as means with standard deviation (SD). Data analysis was performed using GraphPad Prism 5. ANOVA (one- or two-way) with Bonferroni as posthoc test (*p* < 0.05) was used for statistical comparison.

## 3. Results and Discussion

### 3.1. Synthesis of Insulin INS-SDS Ionic Complex

**Figure 1:**
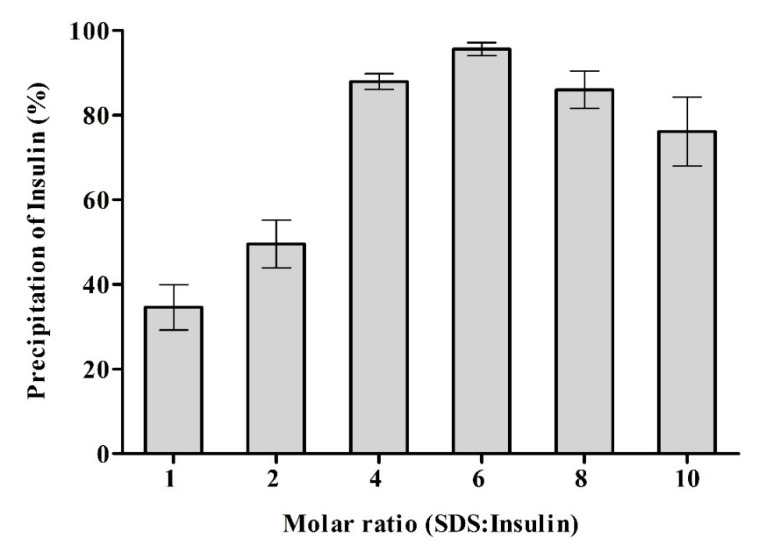
Precipitation efficiency of INS with indicated molar ratios of sodium dodecyl sulfate (SDS). A total of 1 mM INS solution in 0.01 M HCl was mixed with aqueous SDS solution at indicated molar ratios. Resulting INS-SDS complexes were obtained as precipitates that were separated by centrifugation. The remaining free INS present in the supernatant was quantified via HPLC to calculate precipitation efficiency at each molar ratio. Data are shown as means ± SD (*n* = 3).

Sodium dodecyl sulfate (SDS) was used as a lipophilic counter ion to increase the lipophilicity of insulin. SDS was chosen because of its sulfate moiety as sulfonate and sulfate groups were found to be superior over carboxylate groups regarding precipitation efficiency and stability of resulting ionic complexes [19]. INS-SDS complex was formed at various INS to SDS molar ratios ranging from 1:2 to 1:10. As insulin contains six basic amino acids, theoretically, maximum complexation of insulin should occur at INS to SDS molar ratio of 1:6. Results of precipitation efficiency confirmed this theory, as shown in Figure 1. Complexation of insulin decreased at further increasing SDS to INS molar ratios. At INS to SDS molar ratios 1: > 6, not all SDS molecules are bound to counter ions on the peptide. The surplus concentration of SDS having been determined in our experiment at the ratio of 1:10 equals a concentration of 4 mM for free SDS. Although this concentration is below the critical micelle concentration of SDS, nonetheless, the surfactant may form micelles and re-dissolve some ionic complex as described previously for similar complexes [14,20].

**Figure 2:**
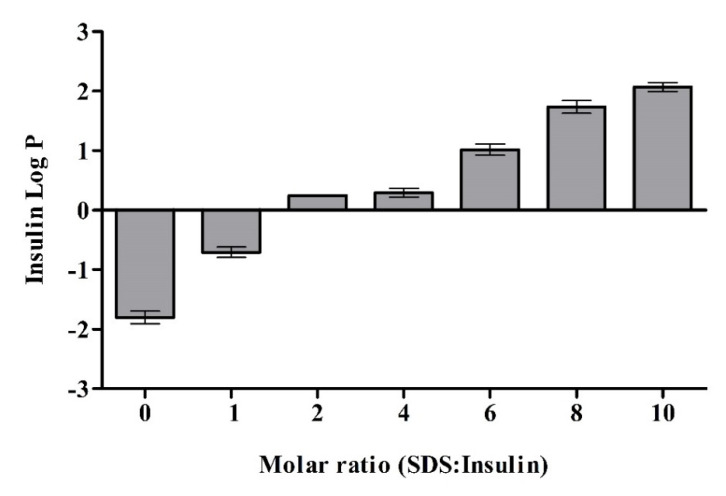
Log P_octanol/water_ of INS with indicated molar ratios of sodium dodecyl sulfate (SDS). INS and INS-SDS complexes were incubated in octanol/water (1:1) at 37 °C, 500 rpm for 12 h. Octanol and water phases were separated by centrifugation, and INS was quantified in each phase via HPLC. Data are shown as means ± SD (*n* = 3).

Lipophilicity of the formed INS-SDS complexes was evaluated by determining log P_octanol/water_ as illustrated in Figure 2. Lipophilicity increase was directly proportional to SDS concentration in INS-SDS complexes. INS-SDS complex formed at INS to SDS molar ratio of 1:10 exhibited the highest lipophilicity and was chosen for further studies with the expectation to achieve high incorporation in NLCs. In a previous study, Muntoni et al. used glargine INS to sodium dodecyl sulfate molar ratio of 1:8 in order to fully cover the charged groups and to achieve high lipophilicity [11]. Lipophilic drugs disperse well in the lipid matrix, whereas hydrophilic drugs are thermodynamically immiscible and separate to the outside of the lipid matrix [21].

### 3.2. NLC Formulations

NLCs were prepared using the solvent diffusion technique as it provides the advantage of heat avoidance during NLC preparation. [22]. In addition, in a previous study, the INS-glargine-sodium dodecyl sulfate complex was successfully loaded in NLCs under similar experimental conditions [11]. NLC preparation methods using higher temperature and shear stress can affect peptide stability [23,24]. Three NLC formulations were prepared using different surfactants, as shown in Table 1.

The percentage of liquid lipids was 30% in all formulations. The usual solid-to-liquid lipid ratio in NLC may vary from 70:30 to 99.9:0.1 [25]. Liquid lipid contributes to an improved drug solubility in the lipid matrix and a higher entrapment efficiency [26]. More spherical and smaller stearic acid NLCs can be obtained at or above 30% liquid lipids [27]. As proteolytic enzymes need an aqueous environment to become active, the type of surfactants present on the lipid/water interface can affect enzyme lipid interactions. Lipases secreted into gastrointestinal lumen target ester linkages [28]. Therefore, ester-free PEG-ether NLCs were designed to improve stability under physiological conditions.

INS-SDS complex loaded NLCs were prepared by dissolving INS-SDS in the organic solvent mixture along with lipids during the preparation of NLCs. Thereafter, entrapment efficiency was calculated based on unentrapped INS present in the supernatant after the separation of NLC aggregates. Results are represented in Table 2.

All blank and INS-SDS complex loaded NLCs were characterized regarding particle size, polydispersity index, and zeta potential. Results are shown in Table 3. After incorporation of INS-SDS, the particle size of PEG-ester and PG-ester NLCs was increased, while in the case of PEG-ether NLC, particle size was slightly decreased. All formulations exhibited negative zeta potential. Incorporation of INS-SDS complex showed no or a minor effect on zeta potential of NLCs. The particle size of lipid nanoparticles is affected by various parameters such as the composition of the formulation and preparation method [22]. As the method to prepare NLCs and percentage of lipids and surfactants was the same in all formulations, the difference in particle size and zeta potential can only be attributed to varying surfactants and lipids of NLCs.

### 3.3. Cytotoxicity

**Figure 3:**
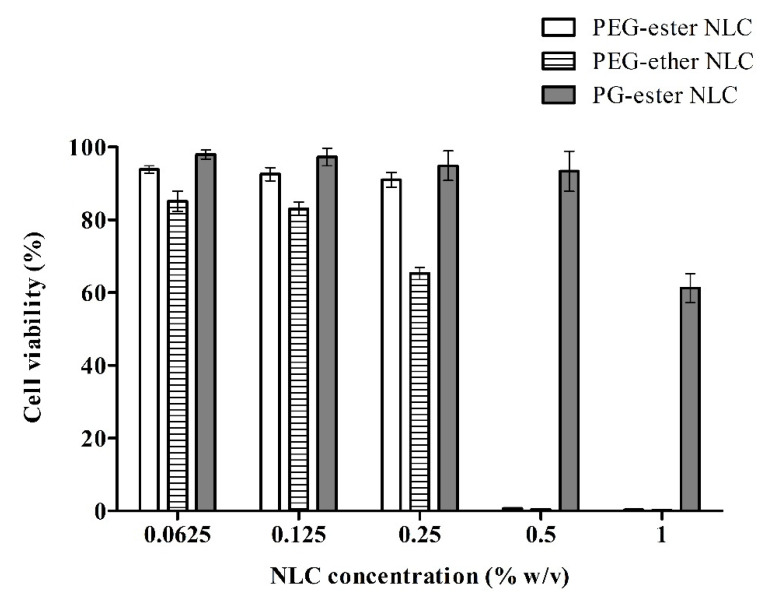
Viability of Caco-2 cells after 4 h incubation with INS-SDS complex loaded NLCs at indicated concentrations determined via resazurin assay. Samples were prepared in 25 mM HBS (HEPES buffered saline) pH 7.4. HBS only was used as a positive control, and Triton X-100 (0.2%, *v*/*v*) in HBS was used as a negative control for cell viability. Data are shown as means ± SD (*n* = 3).

Cytotoxicity evaluation of nanocarrier systems is essential from the safety point of view. As enterocytes are the main cells found in the small intestine [29], the Caco-2 cell line was used to assess the cytotoxicity of NLCs. These cells can differentiate into a monolayer having the same morphology and function as enterocytes [30]. Toxicity was evaluated using a resazurin assay that is based on the oxidation-reduction reaction of the reagent (resazurin) catalyzed by the enzymes of metabolically active viable cells. Cell viability data represented in Figure 3 is for peptide-loaded NLCs. Blank NLC formulations also showed the same cell viability as INS-SDS loaded formulations (data not shown). The concentration at which cell viability was > 80% was considered safe, as reported in previous studies [20,31]. NLCs showed significant differences in cytotoxicity. PG-ester NLCs were found least toxic for Caco-2 cells. The highest safe concentrations were 0.5, 0.25, and 0.125% (*w*/*v*) for PG-ester, PEG-ester, and PEG-ether NLCs, respectively.

PG surfactants are mainly composed of mono- or polyglycerol esterified with fatty acids of variable length. They are reported to have a safe toxicological profile [32], but no study is available providing experimental proof for their lower cytotoxicity compared to PEG-ester or ether-based surfactants. In this study, PEG-ester and PG-ester NLCs differ only in surfactant composition. So it provides evidence for less cytotoxicity of PG-ester surfactants than PEG-ester surfactants.

In addition to variable linkages, the carbon-chain length of surfactants can affect their solubilizing properties and orientation on lipophilic cell membranes. Ionic surfactants show higher cytotoxicity than the non-ionic ones, and cationic surfactants are more harmful than their anionic counterparts due to more intensive interactions with cell membranes. Surfactants may cause disruptions in cellular functions due to interactions with cell membranes and modification of membrane fluidity [31]. So it is important to find the safe concentration at which they do not exert harmful effects on biological membranes. NLCs containing lipids capable of undergoing biodegradation are generally considered biocompatible. However, concentration-dependent toxicity was observed for NLCs probably due to the presence of additional components in the formulation. Stearic acid, oleic acid, and other long-chain fatty acids are also reported to interact with cellular membranes and exert a penetration-enhancing effect [3].

Chemical groups susceptible to enzymatic hydrolysis are mainly esters, and also amides (not all), and probably ketones but not ethers [33]. The highest toxicity of PEG-ether NLC can be related to the absence of ester moieties in the lipids and non-biodegradable ether linkage. Probably they caused non-reversible Caco-2 cells membrane disruptions leading toward increased toxic effects.

### 3.4. Lipolysis

**Figure 4:**
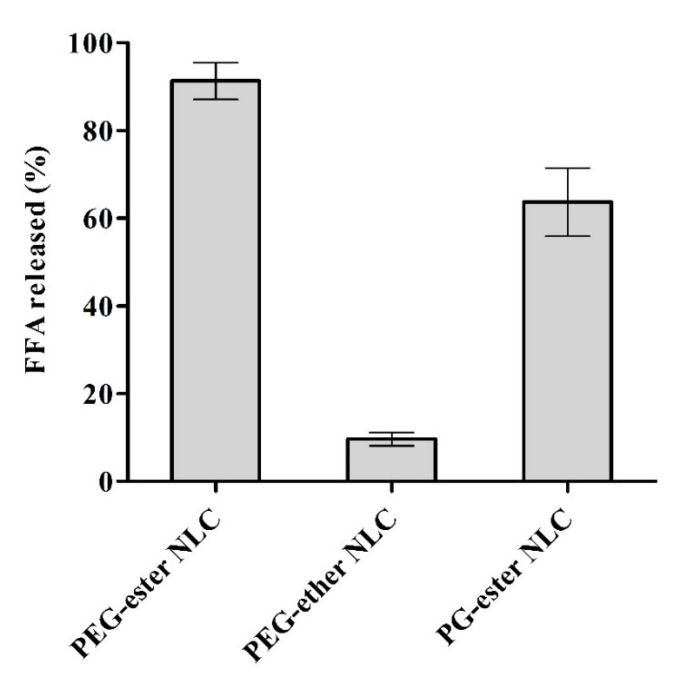
Percent free fatty acid (FFA) released from NLCs during lipolysis study for 60 min. Lipolysis was determined by the pH-stat titration method in lipid digestion buffer containing pancreatin at pH 7.5 and 37 °C. FFA release during the experiment was titrated with 0.5 M NaOH. Data are shown as means ± SD (*n* = 3).

An in vitro lipolysis test was used to quantify the lipid digestion of NLCs. Figure 4 depicts the percentage of free fatty acids released during lipolysis. Lipid composition and surface properties significantly affected the lipolysis behavior of NLCs. The results of lipolysis closely relate to available ester linkages within NLCs. PEG-ether NLCs omitting ester bonds showed resistance to pancreatic lipase, and consequently, higher protection of insulin can be assumed for PEG-ether NLCs. Results are in agreement with a previous study where SEDDS formulation containing no ester substructures were not hydrolyzed by lipase [28]. NLCs having PEG-ester substructures on their surface showed a higher release of free fatty acids in comparison to NLCs having PG-ester surface active moieties, as shown in Figure 4. As both PEG and PG-ester NLCs contain the same solid and liquid lipids, the difference in lipolysis can be attributed to variable hydrolysis rates of PEG and PG esters under the tested experimental conditions.

PEG-ester and PG-ester surfactants also differ in carbon-chain length. Kolliphor RH 40 contains long-chain hydroxystearate, whereas Tegosoft PC41 and Tegosolve 90MB contain medium-chain caprate and caprylate esters. Medium fatty chains of surfactants exhibit probably a higher protective effect against degrading enzymes than long fatty chains. Liu et al. also showed higher protection of INS-phosphatidylcholin complex incorporated in SEDDS containing medium-chain glycerides compared to SEDDS containing long-chain glycerides toward α-chymotrypsin [34]. In another study, octreotide was to a higher extent protected toward enzymatic degradation in the gastrointestinal tract by SEDDS containing long-chain triglycerides than medium-chain triglycerides [35]. The difference in lipolysis of NLCs can be explained further by a difference in the dispersion properties of the formulations due to different surfactants used [36]. The particle size of PG-ester NLCs is also higher than PEG-ester NLCs and can provide more physical shielding of the lipid-water interface from lipase access and reducing the available lipid surface area for enzymatic digestion [37]. In another study, the particle size of SLNs was also indirectly related to the degradation rate [12].

According to literature, the hindrance provided by sterically stabilizing chains of surfactants can also affect the access of lipase to the substrate and consequently the degradation velocity [12]. Arnold et al. showed comparatively slower lipase digestion of PEGylated and polyglycerol surfactants than mono-, di-, and triglycerides of medium-chain fatty acids [38]. PEG coating provides stearic hindrance toward access of lipase to the lipid surface: the more ethylene oxide groups are on the surface of the particle, the more they provide stearic hindrance [39]. Both surfactants used in PEG-ester NLC and PEG-ether NLC contain PEG but differ in the chain length. Kolliphor RH 40 has an average of 13 units of ethylene oxide, while in Brij L23, there are 23 units of ethylene oxide that might cause higher resistance toward lipase access and higher protection. The main difference in Kolliphor RH 40 and Brij L23 surface active agents is the covalent linkage between fatty acids and the hydrophilic PEG substructure. Kolliphor RH 40 bears an ester linkage, while Brij L23 bears an ether linkage. PEG fatty acid esters are potential substrates for digestive lipases [40]. We assume that these chemical linkages on the surfaces of NLCs are easily accessible by lipid digesting enzymes due to their orientation toward the aqueous medium at the lipid-water interface. As esters are degradable by pancreatic lipase, PEG-ester NLCs showed higher lipolysis, and PEG-ether NLCs showed minor lipolysis due to the absence of any ester linkage.

### 3.5. Proteolysis

**Figure 5:**
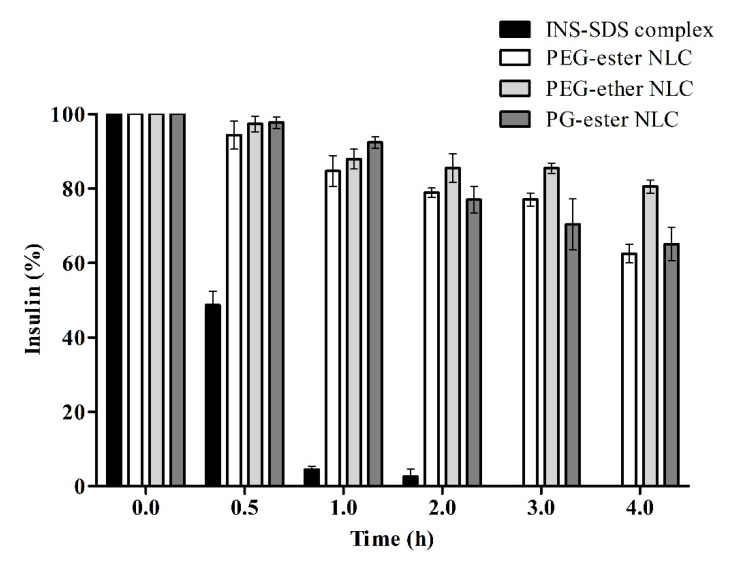
Remaining percentage of intact insulin in indicated formulations compared to the unformulated INS-SDS complex during degradation in SGF at 37 °C and pH 1.2. At each time point, pepsin activity in withdrawn aliquots was quenched by the addition of 0.002 M NaOH. Data are shown as means ± SD (*n* = 3).

Longer peptides such as INS are considered to be more susceptible to degrading enzymes due to the higher number of cleavage sites available than in the case of smaller peptides [18]. The stability of insulin in SGF containing pepsin is shown in Figure 5. A significant amount of insulin remained intact even after 4 h of incubation. PEG-ether NLCs showed the highest protection.

**Figure 6:**
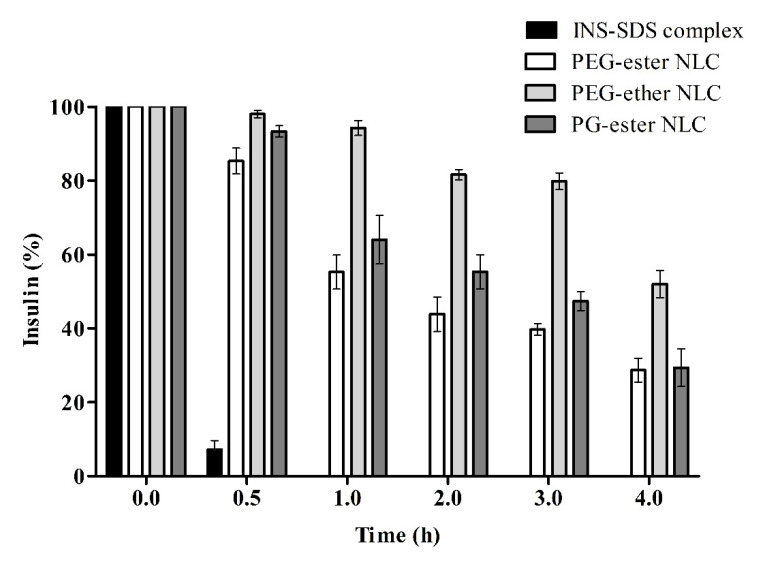
Remaining percentage of intact insulin in indicated formulations compared to the unformulated INS-SDS complex during degradation in SIF at 37 °C and pH 6.8. At each time point, pancreatin activity in withdrawn aliquots was quenched by the addition of 0.01 M HCl. Data are shown as means ± SD (*n* = 3).

Pancreatin contains various enzymes that can be involved in the degradation of lipid nanocarriers as well as peptides. Therefore, the stability of insulin was also investigated in SIF-containing pancreatin. Results are shown in Figure 6. A comparatively higher degradation of INS was observed in SIF than in SGF. This observation might be explained by the presence of lipase in pancreatin in the case of SIF. The highest protective effect of PEG-ether NLCs in comparison to others can be explained by the lack of ester substructures on surfaces susceptible to lipid digesting enzymes of pancreatin. As enzymes cannot enter into the lipid phase, the incorporated peptide is protected from proteolytic cleavage.

Proteolysis studies showed that 30–50% of peptides remain intact even after 4 h upon pancreatin degradation of INS-SDS loaded NLCs in comparison to INS-SDS complex that was completely degraded. Insulin loaded into SLNs containing soy lecithin and polyvinyl alcohol as surfactants remained intact by 30% and 50% after 1 h incubation with trypsin and pepsin, respectively [41]. In addition to susceptible ester linkages, surface erosion can also lead to lipid digestion [42]. Different surfactants present on the surface of NLCs can lead to variable rates of surface erosion and consequently proteolytic cleavage of incorporated peptides. All INS-SDS-loaded NLCs showed significantly higher protection of INS than INS-SDS complex dispersion. No INS was detectable after 2 h incubation of INS-SDS complex in SGF and after 0.5 h incubation of INS-SDS complex in SIF.

## 4. Conclusions

INS-SDS complex was successfully loaded into NLCs exhibiting different surface decorations. Lipolysis study revealed higher lipid hydrolysis of NLCs having PEG-ester, intermediate hydrolysis of NLCs having PG-ester, and almost no hydrolysis of ester-free PEG-ether surface active moieties. Cleavable substructures of surfactants on the surface of NLCs appeared to play a crucial role in the overall protective effect of NLCs toward GIT enzymes. NLCs having no susceptible linkages for degrading enzymes can guarantee a strong protective effect for incorporated peptides, although they are less biocompatible. PG-ester surfactant was found superior regarding cytotoxicity profile compared to PEG-ester and PEG-ether surfactants. From the formulation point of view, it is challenging to design lipid nanocarriers with excipients that provide protection to incorporated peptides on their way to the absorption membrane without toxic risk for the biological system.

## Figures and Tables

**Table 1:** Lipid components and surfactants used for NLC formulations.

NLCs	Constituents		S:L Ratio	Surfactant (% *w*/*v*)
**PEG-ester NLC**	Solid lipid (S)	Stearic acid	70:30	1.0
	Liquid lipid (L)	Glyceryl monooleate (Type 40) NF (Peceol^TM^)		
	Surfactant	Polyoxyl hydrogenated castor oil (Kolliphor^®^ RH 40)		
**PEG-ether NLC**	Solid lipid (S)	Stearic acid	70:30	1.0
	Liquid lipid (L)	Oleic acid		
	Surfactant	Polyoxyethylene-23-laurylether (Brij^TM^ L23)		
**PG-ester NLC**	Solid lipid (S)	Stearic acid	70:30	1.0
	Liquid lipid (L)	Glyceryl monooleate (Type 40) NF (Peceol^TM^)		
	Surfactants	Polyglyceryl-4 Caprate (Tegosoft^®^ PC41) and Polyglyceryl-6 Caprylate (Tegosolve^®^ 90MB)		

**Table 2:** Amount of INS-SDS complex having been added during the NLCs preparation process and resulting entrapment efficiency (% *w*/*w*). Data indicated as means ± SD (*n* = 3).

NLCs	INS-SDS Having Been Added during Preparation Process (mg)	Entrapment Efficacy (%)
**PEG-ester NLC**	10	61.5 ± 2.2
**PEG-ether NLC**	10	58.6 ± 3.0
**PG-ester NLC**	10	73.0 ± 6.3

**Table 3:** Characterization of different NLC formulations regarding size, polydispersity index (PDI), and zeta potential. Data indicated as means ± SD (*n* = 3).

Formulations	Blank NLCs			INS-SDS Complex Loaded NLCs		
Parameters	Size (nm)	PDI	Zeta Potential (mV)	Size (nm)	PDI	Zeta Potential (mV)
**PEG-ester NLC**	64.3 ± 2.6	0.5	−11.3	99.3 ± 1.3	0.4	−17.4
**PEG-ether NLC**	143.4 ± 17.0	0.5	−20.8	134.4 ± 3.3	0.2	−19.6
**PG-ester NLC**	121.8 ± 3.9	0.2	−2.2	217.3 ± 8.4	0.3	−18.3
